# Diffusion Equation-Assisted Markov Chain Monte Carlo Methods for the Inverse Radiative Transfer Equation

**DOI:** 10.3390/e21030291

**Published:** 2019-03-18

**Authors:** Qin Li, Kit Newton

**Affiliations:** Department of Mathematics, University of Wisconsin-Madison, Madison, WI 53705, USA

**Keywords:** multi-level MCMC, radiative transfer equation, inverse problems, diffusion limit

## Abstract

Optical tomography is the process of reconstructing the optical properties of biological tissue using measurements of incoming and outgoing light intensity at the tissue boundary. Mathematically, light propagation is modeled by the radiative transfer equation (RTE), and optical tomography amounts to reconstructing the scattering coefficient in the RTE using the boundary measurements. In the strong scattering regime, the RTE is asymptotically equivalent to the diffusion equation (DE), and the inverse problem becomes reconstructing the diffusion coefficient using Dirichlet and Neumann data on the boundary. We study this problem in the Bayesian framework, meaning that we examine the posterior distribution of the scattering coefficient after the measurements have been taken. However, sampling from this distribution is computationally expensive, since to evaluate each Markov Chain Monte Carlo (MCMC) sample, one needs to run the RTE solvers multiple times. We therefore propose the DE-assisted two-level MCMC technique, in which bad samples are filtered out using DE solvers that are significantly cheaper than RTE solvers. This allows us to make sampling from the RTE posterior distribution computationally feasible.

## 1. Introduction

Optical tomography is a medical imaging technique in which near infrared light is sent into biological tissue, and the reflected and transmitted outgoing light at the surface of the tissue is measured [1,2,3,4]. Using information about the incoming and outgoing light, one can determine the properties of the tissue. The use of low-energy light makes optical tomography cheaper and less invasive than traditional methods such as X-ray imaging, although the reconstruction of the properties of the tissue can be more difficult than when high-energy photons are used. Optical imaging can be used to study and monitor many different kinds of tissue, including brain, breast, and joint imaging, as well as monitoring blood oxygenation [5,6,7].

The process of collecting information about the incoming and outgoing light and using it to reconstruct the tissue’s properties is an inverse problem. There are two associated forward models that both map the incoming data (light intensity sent into the tissue) to the outgoing data (light intensity measured coming off the tissue). The first forward model corresponds to the radiative transfer equation (RTE) and is called the albedo operator. Using the albedo operator, the inverse problem may be solved to obtain the scattering coefficient in the RTE. The second forward model corresponds to the diffusion equation (DE) and is called the Dirichlet-to-Neumann (DtN) map. Using the DtN map, one may similarly solve the inverse problem and reconstruct the diffusion coefficient in the DE. Typically, RTE is used for high-energy photons and DE is used for low-energy photons. The energy of photons determines the “mean-free-path” of free transport, and then further determines the strength of scattering. Mathematically, it can be characterized by the Knudsen number, and one can show that in the small Knudsen number regime, the two equations are equivalent, as will be made clearer in Section 3. It was shown in [8] that the coefficients of the RTE are uniquely recoverable in 3D when the entire albedo operator is known, and it was shown in [9] that this reconstruction is Lipschitz stable. On the other hand, using the DtN map to reconstruct the corresponding coefficients of the DE is the famously ill-posed Calderón problem. The uniqueness of the reconstruction has been presented in [10], and the logarithmically ill-posed nature of the problem has been proved in [11].

These two forward models are related, as we will describe. The RTE describes light propagating through a material with some optical properties, here taken to be the scattering coefficient of the material. Let f(x,v) denote the distribution of particles at location *x* with direction *v*, where $x\in \Omega \subset R^{d}$, and $v\in S^{d-1}$, the unit sphere in $R^{d}$, where *d* is the dimension of the problem. In other words, all particles are taken to move with constant unit speed. In the later parts of the paper, we use “velocity” and “direction" interchangeably when no ambiguity is present. For simplicity, we work with a version of the RTE in which the scattering coefficient σ(x) depends only on position. Then the RTE is

$$\begin{array}{cc} v\cdot \nabla f(x,v) & =\sigma (x)Lf(x,v)\;, \end{array}$$

where L is an integral operator describing photons colliding with the media and scattering off. Its explicit formulation is in Section 3.

The diffusion equation, on the other hand, is a simplified model from the RTE, accurate for low-energy photons in the high-scattering, low-absorption regime. Since the scattering is very strong, the distribution achieves equilibrium in the velocity domain, and the light intensity, also known as fluence, becomes a function only on physical space. Suppose that ρ(x) is the light intensity at position *x*, and a(x) is the diffusion coefficient (corresponding to $\frac{1}{\sigma (x)}$ from the RTE, as will be shown in Theorem 2). Then the DE is

$$\begin{array}{cc} C\nabla \cdot \left(a(x)\nabla \rho (x)\right) & =0\;, \end{array}$$

where *C* is a constant depending on dimension. In this case, the map from the Dirichlet data (light intensity or fluence injected into the tissue) to the Neumann data (light propagating out) is used to reconstruct the diffusion coefficient a(x). This map is known as the Dirichlet-to-Neumann (DtN) map.

There is a relationship between the two forward models RTE and DE. One would like to understand, physically and mathematically, why the stability of each model is different in the inverse setting. It turns out that if a(x) and σ(x) satisfy certain relations, the RTE and DE are asymptotically close in the near infrared case. This is made precise below in Theorem 2. Physically, in the forward setting, high-energy photons experience little scattering before exiting the domain, whereas low-energy photons scatter frequently in the tissue before they exit. As a result, the reconstruction of the tissue using high-energy photons is generally crisp, whereas low-energy photons provide more blurred images. We use the “Knudsen number” ϵ to quantify how much scattering a photon experiences in the material. In the low-energy regime, scattering increases, the Knudsen number shrinks to zero, and the RTE converges to the DE in the forward setting. On the inverse side, the inverse problem for the RTE converges to the inverse problem for the DE, meaning that the information carried in the albedo operator is almost the same as that in the DtN map, and therefore the reconstruction of the tissue properties converges in this limit as well. This has been numerically observed in [12,13], and further proved rigorously in [14,15].

A Bayesian solution to the inverse problem is seen as the posterior distribution for the quantity of interest (QoI), in our case the scattering coefficient in the RTE and the diffusion coefficient in the DE. Bayesian methods are particularly useful for inverse problems, because noise in the measurement as well as prior information about the QoI is taken into account naturally [16]. If we observe some noisy data in order to obtain information about the QoI, the solution of the inverse problem is the probability distribution of the QoI given this data, known as the posterior distribution [17]. Bayes’ theorem allows us to determine this distribution given a guess for the probability distribution of the QoI (the prior distribution) and the probability distribution for the data given the QoI (the likelihood function, obtainable from the forward model). As such, there is a sharp distinction between this probabilistic view and other deterministic tools, and the Bayesian formulation gives one the ability to “regularize” an inverse problem with some prior knowledge.

While we theoretically have the posterior distribution in hand, accessing concrete information about it such as its mean and variance can be challenging. One way to obtain this information is by creating a list of samples from the distribution by some means, such as the Markov chain Monte Carlo (MCMC) method. Even this, however, is not always straightforward. One sample from the RTE represents an entirely new configuration of the media, meaning that one must re-compute the forward albedo map for each MCMC sample. This is especially expensive because the RTE is over phase space: if the domain is three-dimensional, then *f* is supported on $\left(x,v\right)\in \Omega \times S^{d-1}$, a five-dimensional space. The same process applies for the DE as well, but since the DE is only over physical space, these computations are much faster. Knowing that the RTE converges to the DE in the high scattering regime, one wishes to combine the two and speed up the computation.

There is a balance to be struck here, and the goal of this paper is to find a way to sample from the posterior distribution for σ in a way that combines the DE and RTE. To that end, we employ the two-level MCMC method [18], also known as the two-stage method. The two-level method uses two models, which we will call the low-resolution model and the high-resolution model. The high-resolution model gives rise to the desired target distribution—for us, the posterior distribution for the RTE. The low-resolution model gives rise to a distribution that approximates the target distribution, and is ideally fast to compute. It is used to filter out poor draws so we need not waste time evaluating the target distribution on them. We know the DE is fast to compute, and that when scattering is sufficiently strong, the posterior based on the DE is a good approximation to the posterior based on the RTE, so we use the posterior based on the DE as the low-resolution model. Then, we can use the DE to reduce the number of times we must solve the RTE by rejecting bad draws. This method combines the inverse problem for the DE and the inverse problem for the RTE to create a faster method for sampling from the inverse problem for the RTE in the diffusion limit.

There are similar methods to approach related problems, such as the multilevel Monte Carlo method for path simulation [19] or the multilevel Monte Carlo method for parametric integration [20]. These methods also combine low-resolution models with a high-resolution model. The algorithm our method relies on, introduced in [18], is similar to the methods proposed in [21,22]. In this view, our algorithm is a two-stage method that increases the number of MCMC iterations for a given computational cost. It uses a two-stage delayed acceptance, in which the candidate sample *y* has to be accepted with a low-resolution model before it is passed on to be either accepted or rejected by the high-resolution model. However, typically for these methods, a coarse-grid approximation is used for the low-resolution model [23]. No matter how coarse the grids are for the RTE, however, they are still on phase space and the dimensionality issue is still left open. In our method, however, a completely different model is used: the inverse diffusion equation is used as a low-resolution model for the inverse radiative transfer equation.

This paper is organized as follows. In Section 2, we provide some necessary background, including a discussion of Bayesian methods for inverse problems, as well as an introduction to Markov chain Monte Carlo methods. In Section 3, we discuss the diffusion limit of the radiative transfer equation in the forward setting, and go on to discuss the inverse setting convergence of the posterior distributions. In Section 4, we discuss the DE-assisted two-level MCMC method, and prove its convergence. We also discuss the dependence of the second-level acceptance rate on ϵ, the Knudsen number. In Section 5, we present our numerical evidence. In particular, we show the convergence of the two forward models, the convergence of the posterior distribution functions quantified using the Hellinger distance, and the improvement of the DE-assisted two-level MCMC over the standard MCMC.

## 2. Background

In this section, we present preliminaries for studying the inverse problem for the RTE in the diffusion limit. In particular, we will first review basic concepts of the Bayesian formulation, and then review the previous algorithms on which our algorithm is based.

These results will be used to study the convergence of the posterior distribution with the RTE or the DE as the forward model, which will be used to develop our numerical method that combines the two posterior distributions.

### 2.1. Bayesian Formulation for Inverse Problems

Bayesian inference is a technique for estimating the distribution of some quantity of interest when some measurements are available. It is based on Bayes’ theorem. A given physical problem may be denoted as

(1) $$\begin{array}{c} b=G(\sigma )+\eta \;, \end{array}$$

where G is the forward map that takes a parameter σ to the measurement *b*, with an added noise η. The forward problem is to find b=G(σ) for a given σ, while most problems in practice are naturally inverse problems, meaning that one conducts experiments and obtains data *b*, and tries to use it to reconstruct the quantity of interest σ.

The Bayesian formulation is a method for retrieving information about σ using *b*. It requires knowledge of the following two probability distribution functions $\mu_{0}$ and $\mu_{\mathrm{error}}$ ahead of time,

- without knowledge of the measurement *b*, a priori σ obeys a certain law,
  $$\sigma \;∼\;\mu_{0}\left(\sigma \right)\;,$$
- and the noise is distributed as:
  $$\eta =b-G\left(\sigma \right)\;∼\;\mu_{\mathrm{error}}\left(\eta \right)\;.$$

In many cases, both distribution functions can be assumed to be normal, i.e., to have a Gaussian-type density function. Suppose that $\mu_{0}$ is a Gaussian distribution concentrated at $m_{0}$ with covariance $C_{0}$ and the noise is concentrated at zero with covariance $C_{\mathrm{error}}$. If σ is finite dimensional then we may express the prior $\mu_{0}$ and likelihood $\mu^{\sigma }$ as follows:

$$\left\{\begin{array}{c} \mu_{0}\left(\sigma \right)=N\left(m_{0},C_{0}\right)\propto \exp\left(-\frac{1}{2}{\left(\sigma -m_{0}\right)}^{⊤}C_{0}^{-1}\left(\sigma -m_{0}\right)\right)\\ \mu^{\sigma }\left(b\right)=\mu_{\mathrm{error}}\left(\eta \right)\propto \exp\left(-\frac{1}{2}{\left(b-G(\sigma )\right)}^{⊤}C_{\mathrm{error}}^{-1}\left(b-G(\sigma )\right)\right) \end{array}\right.\;.$$

Analogous formulae are also available in the infinite dimensional setting; see [16,17] and the references therein.

With this, the posterior distribution of σ, under the condition that *b* is obtained in experiment, is given by Bayes’ theorem,

(2) $$\begin{array}{cc} \mu^{b}\left(\sigma \right) & =\frac{1}{Z}\mu_{0}\left(\sigma \right)\mu^{\sigma }\left(b\right)\\ & =\frac{1}{Z}\exp\left(-\frac{1}{2}{\left(\sigma -m_{0}\right)}^{⊤}C_{\mathrm{prior}}^{-1}\left(\sigma -m_{0}\right)-{\left(b-G(\sigma )\right)}^{⊤}C_{\mathrm{error}}^{-1}\left(b-G(\sigma )\right)\right)\;, \end{array}$$

where

$$Z=\int \mu^{\sigma }\left(y\right)d\mu_{0}\left(\sigma \right)$$

is the normalization factor.

In optical tomography, there are two fundamental models for describing light propagation: the radiative transfer equation (RTE), and the diffusion equation (DE). They rely on the albedo operator and the DtN map for the reconstruction. In the optically thick case (i.e., when photons scatter frequently), the two models are asymptotically close in some sense,

$$\begin{array}{c} G_{\mathrm{RTE}}\left(\sigma \right)\;\approx \;G_{\mathrm{DE}}\left(\sigma \right)\;. \end{array}$$

Correspondingly, the posterior distributions given by the two models are close to each other [24], meaning:

$$\mu_{\mathrm{DE}}^{b}\left(\sigma \right)\;\approx \;\mu_{\mathrm{RTE}}^{b}\left(\sigma \right)\;.$$

To be more precise, there are multiple ways to quantify the distance between two distribution functions. Typically, this distance is quantified by the Kullback–Leibler (KL) divergence or the Hellinger distance [25]. For any two distributions μ and $\mu^{\prime }$ supported on the same space, they are defined by the following,

- KL divergence
  $$\begin{array}{c} d_{\mathrm{KL}}\left(\mu ,\mu^{\prime }\right)=\int \log\frac{\mu }{\mu^{\prime }}d\mu \;, \end{array}$$
- Hellinger distance
  $$\begin{array}{c} d_{\mathrm{Hell}}{\left(\mu ,\mu^{\prime }\right)}^{2}=\frac{1}{2}\int {\left(\sqrt{\frac{d\mu }{d\lambda }}-\sqrt{\frac{d\mu^{\prime }}{d\lambda }}\right)}^{2}d\lambda \;, \end{array}$$

where λ is any pre-chosen distribution function. The Hellinger distance is invariant to the choice of λ. Again, these formulae have interpretations in the infinite dimensional setting; see the Appendix of [17]. In Theorem 3, we quantify the similarity between $\mu_{\mathrm{DE}}^{b}\left(\sigma \right)$ and $\mu_{\mathrm{RTE}}^{b}\left(\sigma \right)$ and find that the two distributions are ϵ apart in the diffusion limit. Therefore, since $\mu_{\mathrm{DE}}^{b}$ is close to $\mu_{\mathrm{RTE}}^{b}$ in the optically thick case, the main task in this paper is to use $\mu_{\mathrm{DE}}^{b}$ to sample from $\mu_{\mathrm{RTE}}^{b}$.

### 2.2. The Markov Chain Monte Carlo Method

We first present the standard Metropolis–Hastings (MH) algorithm. Given a probability density μ called the target distribution and defined on *X*, the MH algorithm constructs a Markov chain on *X* that is stationary with respect to μ. The elements of the Markov chain are then regarded as samples from the distribution μ. More specifically, the MH algorithm starts with an initial guess $x_{0}$, and draws new samples according to a proposal distribution *q*. By adjusting the acceptance rate using the target distribution μ, the MH algorithm accepts or rejects the draws so that the accepted samples form an empirical distribution that resembles the target distribution μ. We now present the MH algorithm, shown in Algorithm 1.

**Algorithm 1** Metropolis Hastings
Given $x_{k}$, draw $y∼q(\cdot ,x_{k})$.Let
$$\alpha \left(x_{k},y\right):=\min\left\{1,\frac{q\left(y,x_{k}\right)\mu \left(y\right)}{q\left(x_{k},y\right)\mu \left(x_{k}\right)}\right\}.$$With probability α, **accept** *y* and set $x_{k+1}=y$. Otherwise, set $x_{k+1}=x_{k}$.


The transition kernel *P* of this algorithm is

$$\begin{array}{c} P\left(x_{k},A\right)=P\left\{x_{k+1}\in A|x_{0},\cdots ,x_{k}\right\}\;. \end{array}$$

In order to demonstrate the convergence of MCMC in Theorem 1, it is necessary to examine the transition kernel, which is the probability that the next draw $x_{k+1}$ is in the set *A* given the previous elements of the chain $x_{0},\cdots ,x_{k}$. It may be written

$$\begin{array}{c} P\left(x,y\right)=p\left(x,y\right)+\delta_{y}\left(x\right)r(x)\;. \end{array}$$

where the off-diagonal density of the kernel is

(3) $$p\left(x,y\right)=\left\{\begin{array}{cc} q(x,y)\alpha (x,y)\; & \;\mathrm{if}\;x\neq y\\ 0\; & \;\mathrm{if}\;x=y \end{array}\right.\;,$$

and the probability that the process remains at *x* is

$$\begin{array}{cc} r(x) & =1-\int p(x,y)dy\;. \end{array}$$

The goal is to show that the target distribution μ is an invariant distribution of the Markov chain ${\left\{x_{k}\right\}}_{k\geq 0}$ in the sense that all measurable sets *A* satisfy

(4) μ(A)=∫μ(x)P(x,A)dx.

This means that elements of the Markov chain generated by Algorithm 1 give a good representation of the distribution μ. To show that μ is the invariant distribution, one first needs p(x,y) to satisfy the detailed balance lemma.

**Lemma** **1.** *The off-diagonal density p(x,y) satisfies the following equation known as “detailed balance",*

(5) μ(x)p(x,y)=μ(y)p(y,x).

This lemma allows us to show the following theorem.

**Theorem** **1.** *The target distribution μ is an invariant distribution of the Markov chain $x_{n}$ with transition kernel P, i.e.,*

(6) μ(A)=∫P(x,A)μ(x)dx.

For our problem, each new proposal *y* is a new configuration of the media σ(x). Thus, to compute $\alpha (x_{k},y)$, one must evaluate μ(y) and thus re-compute the forward map, which requires solving the RTE many times. As this is computationally prohibitive, we instead use the two-level MCMC method, described in the next section. The cost comparison is discussed further in Section 5.

### 2.3. Two-Level MCMC Method

The two-level MCMC method is a method to increase the efficiency of sampling from the target distribution μ. It requires two distributions: a target distribution μ, and a second distribution $\mu^{\prime }$. Here, the target distribution μ calls for the evaluation of a “high-resolution” model, but if it can be approximated in some sense by $\mu^{\prime }$ which only calls for the evaluation of some “low-resolution” model, then $\mu^{\prime }$ can serve as a good filter to reject poor draws in the MCMC sampling. More specifically, the algorithm goes through two levels of evaluating a proposed sample. First, the sample is evaluated using the low-resolution model and is either accepted or rejected. If it is accepted, the algorithm goes on to evaluate the sample using the high-resolution model. This “pre-acceptance” stage filters out poor draws, allowing one to make more courageous proposals and not waste time evaluating the forward model on them. We present the two-level scheme below in Algorithm 2.

**Algorithm 2** Two-level Metropolis Hastings
Given $x_{k}$, draw $y∼q(\cdot ,x_{k})$Let
$$\alpha \left(x_{k},y\right):=\min\left\{1,\frac{q\left(y,x_{k}\right)\mu^{\prime }\left(y\right)}{q\left(x_{k},y\right)\mu^{\prime }\left(x_{k}\right)}\right\}\;.$$With probability α, **pre-accept** *y* and continue to 4. Otherwise, set $x_{k+1}=x_{k}$ and start over.The second-level proposal is now *y*, effectively drawn from
$$q_{2}\left(y,x_{k}\right)=\alpha \left(x_{k},y\right)q\left(y,x_{k}\right)+\delta_{x_{k}}\left(y\right)\left(1-\int \alpha \left(x_{k},y\right)q\left(y,x_{k}\right)dy\right)\;.$$Then set
$$\beta \left(x_{k},y\right):=\min\left\{1,\frac{q_{2}\left(y,x_{k}\right)\mu \left(y\right)}{q_{2}\left(x_{k},y\right)\mu \left(x_{k}\right)}\right\}\;.$$With probability $\beta (x_{k},y)$, **accept** and set $x_{k+1}=y$. Otherwise, set $x_{k+1}=x_{k}$.


Similarly to the MCMC method (Algorithm 1), in the two-level MCMC method, the draw of $x_{k+1}$ merely depends on the evaluation of $x_{k}$, and the previous draws $x_{1},\cdots x_{k-1}$ are irrelevant. The transition kernel that brings $x_{k}$ to $x_{k+1}$ is denoted by $P_{2}$, which we will discuss in more detail in Section 4.1.

In this paper, the desired high-resolution model will be the posterior distribution for the RTE. The low-resolution model will be the posterior distribution for the DE. Evaluating $\mu^{\prime }\left(y\right)$ only involves computing the DE, which is much faster since the DE is supported on the physical domain. Once the DE accepts the proposal *y*, it passes to the inverse problem for the RTE’s posterior distribution μ(y). Thus, we compute the RTE forward model fewer times overall, which saves time.

In Section 4, we discuss the convergence of this method and the dependence of the second-level acceptance rate β on the Knudsen number ϵ, our limit parameter.

## 3. Diffusion Limit

In this section, we examine the diffusion limit of our problem in the forward and inverse case. We first study the convergence of the radiative transfer equation to the diffusion equation. Then, we explain how the inverse problems are solved using the forward map. Next, we discuss the convergence of the forward map for the radiative transfer equation to that of the diffusion equation. Finally, we discuss the convergence of the posterior distribution for the radiative transfer equation to the posterior for the diffusion equation.

### 3.1. Diffusion Limit of the Radiative Transfer Equation

The optical “thickness” of the material physically corresponds to the number of times a photon scatters between entering a medium and escaping. The physical quantity is termed the Knudsen number, which stands for the ratio of mean free path to the domain length. The mean free path is the average distance a photon travels before being scattered. When the Knudsen number is small, photons, on average, scatter many times before they are emitted, and the material is thus regarded as optically “thick”. In this regime, the two mathematical models for light propagation carry the same information, namely, the radiative transfer equation and the diffusion equation are asymptotically converging.

The radiative transfer equation takes a statistical mechanics viewpoint, and describes the distribution of photons on the phase domain. Let f(x,v) denote the number of photons at position $x\in \Omega \subset R^{d}$, a bounded domain, moving in direction $v\in S^{d-1}$, the unit sphere in $R^{d}$ (i.e., the speed is normalized to be 1). This distribution satisfies the RTE,

(7) $$\begin{array}{c} v\cdot \nabla f(x,v)=\sigma (x)Lf(x,v)\;, \end{array}$$

where the collision operator is

(8) $$\begin{array}{c} Lf=\int f\left(x,v^{\prime }\right)dv^{\prime }-f={\langle f\rangle }_{v}-f\;. \end{array}$$

In the equation, the term v·∇f on the left shows that the photons move with direction *v*, and the term on the right shows that the photons colliding with the media and being scattered. We have used the notation ${\langle \rangle }_{v}$ to denote normalized integration over *v*, and dv is the normalized unit measure, meaning

$$\int_{S^{d-1}}1dv=1\;.$$

The equation has a unique solution when it is equipped with the incoming boundary condition, which is the analogue of a Dirichlet boundary condition for equations lacking velocity space. Let

$$\begin{array}{c} \Gamma_{\pm }=\left\{\left(x,v\right):x\in \partial \Omega ,\pm v\cdot n_{x}>0\right\}\; \end{array}$$

denote the collection of coordinates on the boundary x∈∂Ω, so that the velocity *v* points in/out of the domain: $\pm v\cdot n_{x}>0$. Here, $n_{x}$ is the normal vector at *x* pointing out of Ω. The incoming boundary condition is imposed on $\Gamma_{-}$, whereas $\Gamma_{+}$ represents the particles going out of Ω. For a unique solution to (7), boundary conditions must be imposed on $\Gamma_{-}$,

$${f|}_{\Gamma_{-}}=\phi \left(x,v\right)\;.$$

**Remark** **1.** *In fact, a more general model of the radiative transfer equation is*

$$\begin{array}{c} v\cdot \nabla_{x}f\left(x,v\right)=\int k\left(x,v,v^{\prime }\right)f\left(x,v^{\prime }\right)dv^{\prime }-\sigma_{a}\left(x,v\right)f\left(x,v\right)\;. \end{array}$$

*It concerns the case when the scattering coefficient, now seen as $k(x,v,v^{\prime })$, may depend on the changing velocity of the particles during a collision, and also includes an absorption coefficient $\sigma_{a}\left(x,v\right)$ representing the photons being absorbed into the material and lost. A standard k which takes into account other kinds of scattering is given by the Henyey–Greenstein model. The absorption coefficient can be taken to be zero if scattering is sufficiently high, and this is the case we focus on.*

The equation is asymptotically equivalent to the diffusion equation in the optically thick regime when the Knudsen number is small. We denote the Knudsen number by ϵ and rescale the problem by setting σ→σ/ϵ. Then, as the Knudsen number becomes small, the scattering effect dominates. Equation (7) may then be written as

(9) $$\begin{array}{c} \left\{\begin{array}{c} v\cdot \nabla f=\frac{1}{\epsilon }\sigma Lf\;,\;\left(x,v\right)\in \Omega \times S^{d-1}\\ {f|}_{\Gamma_{-}}=\phi \left(x,v\right) \end{array}\right.\;. \end{array}$$

In the small ϵ regime, it was conjectured in [26] and then proved in [27,28] that the equation is asymptotically equivalent to the diffusion equation. One can make the convergence explicit under the following assumptions.

**Assumption** **1.** *Both the media and the boundary conditions are bounded.*

- *the admissible media is bounded, meaning that there is a constant $C_{*}$ so that:*
  $${\max\{∥\sigma ∥}_{L_{\infty }\left(\Omega \right)}\;,∥\sigma^{-1}{∥}_{L_{\infty }\left(\Omega \right)}\;,∥\nabla \left(\sigma^{-1}\right){∥}_{L_{\infty }\left(\Omega \right)}\}<C_{*}\;;$$
- *and the boundary conditions are bounded, meaning:*
  $${\max\{∥\xi ∥}_{L_{\infty }\left(\partial \Omega \right)}{\;,∥\phi ∥}_{L_{\infty }\left(\Gamma \right)}\}<C_{*}\;.$$

*We also term the set of admissible media:*

(10) $$A=\{\sigma \in W_{1,\infty }{\left(\Omega \right):\max\{∥\sigma ∥}_{L_{\infty }\left(\Omega \right)}\;,∥\sigma^{-1}{∥}_{L_{\infty }\left(\Omega \right)}\;,∥\nabla \left(\sigma^{-1}\right){∥}_{L_{\infty }\left(\Omega \right)}\}<C_{*}\}\;.$$

With the assumption, we have the following theorem.

**Theorem** **2.** *Suppose that f(x,v) satisfies Equation (9), then as ϵ→0, f(x,v)→ρ(x), which satisfies*

(11) $$\begin{array}{c} \left\{\begin{array}{c} C_{d}\nabla \cdot \left(\frac{1}{\sigma }\nabla \rho \right)=0,\;x\in \Omega \subset R^{d}\\ \rho {|}_{\partial \Omega }=\xi \left(x\right) \end{array}\right.\;, \end{array}$$

*where ξ(x) is defined by ϕ(x). $C_{d}$ is a constant depending on the dimension d and could be dropped out of Equation (11). In particular, with compatible boundary conditions at different orders, one approximates f through different forms:*

- *if ϕ(x,v)=ξ(x):*
  $${∥f-\rho ∥}_{L_{\infty }\left(\Omega \right)}<C_{A}\epsilon \;.$$
- *if $\phi \left(x,v\right)=\xi \left(x\right)-\epsilon \frac{1}{\sigma }v\cdot \nabla \xi$:*
  $$∥f-\rho +\epsilon \sigma^{-1}{v\cdot \nabla \rho ∥}_{L_{\infty }\left(\Omega \right)}<C_{A}\epsilon^{2}\;.$$

*Here, the constant $C_{A}$ depends on $C_{*}$, the upper bound in Assumption 1 for the admissible set.*

The proof, which we omit here, relies on asymptotic expansion away from the boundary.

### 3.2. Convergence in the Inverse Setting

We examine the convergence in the inverse setting in this section. To describe light propagation in a given tissue in Ω, there are two models, the radiative transfer equation that gives a statistical description, and the diffusion equation that characterizes the macroscopic behavior. The two models are asymptotically equivalent, as discussed in the previous section.

In optical tomography, light with a known intensity is injected into the material, and detectors are placed on the tissue boundary to collect the light current emitted from the material. For the RTE, the mapping from the incoming data to the outgoing data is termed the albedo operator, defined by $H^{\mathrm{RTE}}$,

(12) $$H^{\mathrm{RTE}}\left(\sigma \right):\;\phi \left(x\right)\to h^{\mathrm{RTE}}\left(x\right)=\frac{1}{C_{d}\epsilon }\int v\cdot nf{|}_{\Gamma_{+}}dv\;,$$

where *f* satisfies (9). It may also be written:

(13) $$h^{\mathrm{RTE}}=H^{\mathrm{RTE}}\left(\sigma \right)\phi \;.$$

In practice, finitely many incoming data $\phi_{k}$ are injected and finitely many measurements are taken at the boundary location $l_{j}$ per experiment. We define the map, determined by the to-be-reconstructed σ, from the known incoming data to the measured outgoing data as:

(14) $$b_{j,k}=l_{j}\left(h^{\mathrm{RTE}}\right)+\eta_{j,k}=l_{j}\left(H^{\mathrm{RTE}}\left(\sigma \right)\phi_{k}\right)+\eta_{j,k}\;,\;\left(j,k\right)\in \left\{1,\cdots ,J\right\}⊗\left\{1,\cdots ,K\right\}\;,$$

or in a compact form:

(15) $$\vec{b}=G^{\mathrm{RTE}}\left(\sigma \right)+\vec{\eta }\;,$$

where $\vec{\eta }$ is a vector of JK length that contains the noise in the measurements. Clearly, the JK length vector $\vec{b}$ is the result of a forward map G acting on the quantity of interest σ, with a small perturbation due to the noise. We assume that the noise is distributed as a Gaussian,

(16) $$\vec{\eta }∼N\left(0,\gamma^{2}I\right)\propto \exp\left(-\frac{1}{2\gamma^{2}}\sigma^{2}\right)\;,$$

meaning:

(17) $$\vec{b}|\sigma ∼N\left(G^{\mathrm{RTE}}\left(\sigma \right),\gamma^{2}I\right)\propto \exp\left(-\frac{1}{2\gamma^{2}}{\left(b-G^{\mathrm{RTE}}\left(\sigma \right)\right)}^{⊤}\left(b-G^{\mathrm{RTE}}\left(\sigma \right)\right)\right)\;.$$

According to Bayes’ theorem, one then has:

(18) $$\mu_{\mathrm{RTE}}^{\vec{b}}\left(\sigma \right)=\frac{1}{Z^{\mathrm{RTE}}}\mu_{\mathrm{RTE}}^{\sigma }\left(\vec{b}\right)\mu_{0}\left(\sigma \right)\;.$$

For the DE model, we consider the forward map to be the map that takes the Dirichlet data to the Neumann outflow. It is termed the DtN map:

(19) $$\begin{array}{c} H^{\mathrm{DE}}\left(\sigma \right):\;\xi \left(x\right)\to h^{\mathrm{DE}}\left(x\right)=\frac{1}{\sigma }\frac{\partial \rho }{\partial n}\;, \end{array}$$

where ρ satisfies (11). Another way to write it is:

(20) $$h^{\mathrm{DE}}=H^{\mathrm{DE}}\left(\sigma \right)\xi \;.$$

Again, in practice, finitely many incoming data $\xi_{k}$ are injected and finitely many measurements are taken at the boundary $l_{j}$ per experiment. We define the map, determined by the to-be-reconstructed media σ, from $\xi_{k}$ to the measured data as:

(21) $$b_{j,k}=l_{j}\left(h^{\mathrm{DE}}\right)+\eta_{j,k}=l_{j}\left(H^{\mathrm{DE}}\left(\sigma \right)\phi_{k}\right)+\eta_{j,k}\;,\;\left(j,k\right)\in \left\{1,\cdots ,J\right\}⊗\left\{1,\cdots ,K\right\}\;,$$

or:

(22) $$\vec{b}=G^{\mathrm{DE}}\left(\sigma \right)+\vec{\eta }\;,$$

where $\vec{\eta }$ is the same pollution in the measurement. Again, the vector $\vec{b}$ is the result of the forward map for the diffusion equation acting on the to-be-reconstructed σ, with the perturbation from the noise. Then, similarly, we have

(23) $$\mu_{\mathrm{DE}}^{\vec{b}}\left(\sigma \right)=\frac{1}{Z^{\mathrm{DE}}}\mu_{\mathrm{DE}}^{\sigma }\left(\vec{b}\right)\mu_{0}\left(\sigma \right)\;.$$

It is proved in [24] that the two forward maps converge, namely:

**Proposition** **1.** *Under Assumption 1, the forward maps $G^{\mathrm{RTE}}$ and $G^{\mathrm{DE}}$ satisfy*

$$∥G^{\mathrm{RTE}}\left(\sigma \right)-G^{\mathrm{DE}}{\left(\sigma \right)∥}_{\infty }=O\left(\epsilon \right)\;,$$

*for all σ∈A.*

Using this convergence, it was also proved that the posterior distributions are close in the diffusion limit, as in the following theorem.

**Theorem** **3.** *Under Assumption 1, the Hellinger distance between the two posterior distribution is bounded by ϵ in the optically thick regime when ϵ→0, namely,*

$$d_{\mathrm{Hell}}\left(\mu_{\mathrm{RTE}}^{\vec{b}},\mu_{\mathrm{DE}}^{\vec{b}}\right)=O\left(\epsilon \right)\;.$$

*Similarly, the Kullback–Leibler divergence between the posterior distribution for the RTE and the posterior distribution for the DE is also O(ϵ),*

$$d_{\mathrm{KL}}\left(\mu_{\mathrm{RTE}}^{\vec{b}},\mu_{\mathrm{DE}}^{\vec{b}}\right)=O\left(\epsilon \right)\;.$$

The proof largely depends on the Lipschitz continuity of the Gaussian form in the likelihood function, and the convergence result in Proposition 1. We omit the details and refer interested readers to [24].

Using Theorem 3, when ϵ is sufficiently small, one may use the diffusion equation posterior to approximate the radiative transfer equation posterior. This approximation allows us to speed up the MCMC computation by setting $\mu_{\mathrm{DE}}^{\vec{b}}=\mu^{\prime }$ as the low-resolution model in the first level. This filters out bad draws, passing better draws to $\mu_{\mathrm{RTE}}^{\vec{b}}=\mu$ on the second level.

## 4. Algorithm

In this section, we discuss the DE-assisted two-level MCMC method and its convergence for our case, the inverse problem for the RTE in the diffusion limit. We present a result about the second-level acceptance rate of two-level MCMC and its dependence on ϵ and a result about the computational cost of our algorithm compared to the one-level MCMC method.

### 4.1. DE-Assisted Two-Level MCMC Method

In this section, we discuss our algorithm, the DE-assisted two-level MCMC method. It is shown below in Algorithm 3.

**Algorithm 3** DE-assisted two-level Metropolis Hastings
Given $x_{k}$, draw $y∼q(\cdot ,x_{k})$Let
$$\alpha \left(x_{k},y\right):=\min\left\{1,\frac{q\left(y,x_{k}\right)\mu_{\mathrm{DE}}^{\vec{b}}\left(y\right)}{q\left(x_{k},y\right)\mu_{\mathrm{DE}}^{\vec{b}}\left(x_{k}\right)}\right\}\;.$$With probability α, **pre-accept** *y* and continue to 4. Otherwise, set $x_{k+1}=x_{k}$ and start over.The second-level proposal is now *y*, effectively drawn from
$$q_{2}\left(y,x_{k}\right)=\alpha \left(x_{k},y\right)q\left(y,x_{k}\right)+\delta_{x_{k}}\left(y\right)\left(1-\int \alpha \left(x_{k},y\right)q\left(y,x_{k}\right)dy\right)\;.$$Then set
$$\beta \left(x_{k},y\right):=\min\left\{1,\frac{q_{2}\left(y,x_{k}\right)\mu_{\mathrm{RTE}}^{\vec{b}}\left(y\right)}{q_{2}\left(x_{k},y\right)\mu_{\mathrm{RTE}}^{\vec{b}}\left(x_{k}\right)}\right\}\;.$$With probability $\beta (x_{k},y)$, **accept** and set $x_{k+1}=y$. Otherwise, set $x_{k+1}=x_{k}$.


The transition kernel $P_{2}$ may be written

$$\begin{array}{c} P_{2}\left(x,y\right)=p_{2}\left(x,y\right)+r_{2}\left(x\right)\delta_{x}\left(y\right)\;, \end{array}$$

where

$$\begin{array}{c} p_{2}\left(x,y\right)=\left\{\begin{array}{cc} q_{2}\left(x,y\right)\beta \left(x,y\right) & \;x\neq y\\ 0 & \;x=y \end{array}\right. \end{array}$$

is called the second-level off-diagonal density, and $r_{2}\left(x\right)=1-\int p_{2}\left(x,y\right)dy$. As before, to demonstrate the convergence of MCMC, we will examine the transition kernel, which is the probability that the next draw $x_{k+1}$ is in the set *A* given the previous elements of the chain $x_{0},\cdots ,x_{k}$.

For the two-level MCMC method, one desires that the high-resolution target distribution $\mu_{\mathrm{RTE}}^{\vec{b}}$ is an invariant distribution of the Markov chain ${\left\{x_{k}\right\}}_{k\geq 0}$. By definition, this is true if for all measurable sets *A*,

$$\begin{array}{c} \mu_{\mathrm{RTE}}^{\vec{b}}\left(A\right)=\int \mu_{\mathrm{RTE}}^{\vec{b}}\left(x\right)P\left(x,A\right)dx\;. \end{array}$$

This means that elements of the Markov chain generated by Algorithm 3 give a good representation of the distribution $\mu_{\mathrm{RTE}}^{\vec{b}}$. To show that $\mu_{\mathrm{RTE}}^{\vec{b}}$ is the invariant distribution, we first need the following two lemmas.

**Lemma** **2.** *The second-level acceptance rate may be written*

$$\begin{array}{c} \beta \left(x_{k},y\right)=\min\left\{1,\frac{q_{2}\left(y,x_{k}\right)\mu_{RTE}^{\vec{b}}\left(y\right)}{q_{2}\left(x_{k},y\right)\mu_{RTE}^{\vec{b}}\left(x_{k}\right)}\right\}=\min\left\{1,\frac{\mu_{DE}^{\vec{b}}\left(x_{k}\right)\mu_{RTE}^{\vec{b}}\left(y\right)}{\mu_{DE}^{\vec{b}}\left(y\right)\mu_{RTE}^{\vec{b}}\left(x_{k}\right)}\right\}\;, \end{array}$$

*where $q_{2}$ is defined as in Algorithm 2.*

**Proof.**  From Lemma 3, for $x_{k}\neq y$, we have

$$\begin{array}{cc} \frac{q_{2}\left(y,x_{k}\right)}{q_{2}\left(x_{k},y\right)} & =\frac{\alpha (y,x)q(y,x)}{\alpha (x,y)q(x,y)}\\ & =\frac{\mu_{\mathrm{DE}}^{\vec{b}}\left(x\right)}{\mu_{\mathrm{DE}}^{\vec{b}}\left(y\right)}\;. \end{array}$$

Plugging this in to our definition of β, we find

(24) $$\begin{array}{c} \beta \left(x_{k},y\right)=\min\left\{1,\frac{\mu_{\mathrm{DE}}^{\vec{b}}\left(x_{k}\right)\mu_{\mathrm{RTE}}^{\vec{b}}\left(y\right)}{\mu_{\mathrm{DE}}^{\vec{b}}\left(y\right)\mu_{\mathrm{RTE}}^{\vec{b}}\left(x_{k}\right)}\right\}\;. \end{array}$$

 □

The importance of this lemma is that it equates β, which depends on $q_{2}$, to a form that is computable. Note that $q_{2}$ has a complicated integral form and thus is numerically challenging to compute. This form of the second-stage acceptance rate β has been calculated in [18]. Later, we will discuss the dependence of β on ϵ through the inverse problem for the RTE’s posterior distribution and show that when the RTE and DE are close, β is close to one.

We next discuss a property of β that we will need to show the convergence of the two-level MCMC method.

**Lemma** **3.** *The second-level off-diagonal density satisfies the detailed balance equation*

$$\begin{array}{c} p_{2}\left(x,y\right)\mu_{RTE}^{\vec{b}}\left(x\right)=p_{2}\left(y,x\right)\mu_{RTE}^{\vec{b}}\left(y\right)\;. \end{array}$$

**Proof.**  Considering the form of β in Equation (24), when $\mu_{\mathrm{DE}}^{\vec{b}}\left(x\right)\mu_{\mathrm{RTE}}^{\vec{b}}\left(y\right)<\mu_{\mathrm{DE}}^{\vec{b}}\left(y\right)\mu_{\mathrm{RTE}}^{\vec{b}}\left(x\right)$,

$$\begin{array}{c} \beta \left(x,y\right)=\frac{\mu_{\mathrm{DE}}^{\vec{b}}\left(x\right)\mu_{\mathrm{RTE}}^{\vec{b}}\left(y\right)}{\mu_{\mathrm{DE}}^{\vec{b}}\left(y\right)\mu_{\mathrm{RTE}}^{\vec{b}}\left(x\right)},\;\beta \left(y,x\right)=1\;. \end{array}$$

Similarly, when $\mu_{\mathrm{DE}}^{\vec{b}}\left(x\right)\mu_{\mathrm{RTE}}^{\vec{b}}\left(y\right)>\mu_{\mathrm{DE}}^{\vec{b}}\left(y\right)\mu_{\mathrm{RTE}}^{\vec{b}}\left(x\right)$,

$$\begin{array}{c} \beta \left(x,y\right)=1,\;\beta \left(y,x\right)=\frac{\mu_{\mathrm{DE}}^{\vec{b}}\left(y\right)\mu_{\mathrm{RTE}}^{\vec{b}}\left(x\right)}{\mu_{\mathrm{DE}}^{\vec{b}}\left(x\right)\mu_{\mathrm{RTE}}^{\vec{b}}\left(y\right)}\;, \end{array}$$

so dividing them gives

$$\begin{array}{cc} \frac{\beta (x,y)}{\beta (y,x)} & =\frac{\mu_{\mathrm{DE}}^{\vec{b}}\left(x\right)\mu_{\mathrm{RTE}}^{\vec{b}}\left(y\right)}{\mu_{\mathrm{DE}}^{\vec{b}}\left(y\right)\mu_{\mathrm{RTE}}^{\vec{b}}\left(x\right)}=\frac{\mu_{\mathrm{RTE}}^{\vec{b}}\left(y\right)q_{2}\left(y,x\right)}{\mu_{\mathrm{RTE}}^{\vec{b}}\left(x\right)q_{2}\left(x,y\right)}\;, \end{array}$$

using Lemma 1. Simplifying gives

$$\begin{array}{c} \mu_{\mathrm{RTE}}^{\vec{b}}\left(x\right)q_{2}\left(x,y\right)\beta \left(x,y\right)=\mu_{\mathrm{RTE}}^{\vec{b}}\left(y\right)q_{2}\left(y,x\right)\beta \left(y,x\right)\;. \end{array}$$

 □

This lemma gives rise to the following theorem.

**Theorem** **4.** *The second-level distribution $\mu_{RTE}^{\vec{b}}$ is the invariant distribution of the Markov chain $\left\{x_{n}\right\}$ with second-level transition kernel $P_{2}\left(x,A\right)$, i.e.,*

(25) $$\begin{array}{c} \mu_{RTE}^{\vec{b}}\left(A\right)=\int p_{2}\left(x,A\right)\mu_{RTE}^{\vec{b}}\left(x\right)dx\;, \end{array}$$

*where the transition kernel $p_{2}\left(x,y\right)$ is defined as*

$$\begin{array}{c} P_{2}\left(x,y\right)=p_{2}\left(x,y\right)+r_{2}\left(x\right)\delta_{x}\left(y\right)\;. \end{array}$$

**Proof.**  Consider

$$\begin{array}{cc} \int P_{2}\left(x,A\right)\mu_{\mathrm{RTE}}^{\vec{b}}\left(x\right)dx & =\int p_{2}\left(x,y\right)\mu_{\mathrm{RTE}}^{\vec{b}}\left(x\right)dx+\int r_{2}\left(x\right)\delta_{x}\left(A\right)\mu_{\mathrm{RTE}}^{\vec{b}}\left(x\right)dx\\ & =\int_{A}\int p_{2}\left(y,x\right)\mu_{\mathrm{RTE}}^{\vec{b}}\left(y\right)dxdy+\int_{A}r_{2}\left(x\right)\mu_{\mathrm{RTE}}^{\vec{b}}\left(x\right)dx\;, \end{array}$$

using Lemma 3 and using the delta function to perform the integration over *x* in the second term. Then, from the definition of $p_{2}$,

$$\begin{array}{cc} \int P_{2}\left(x,A\right)\mu_{\mathrm{RTE}}^{\vec{b}}\left(x\right)dx & =\int_{A}\left(1-r_{2}\left(y\right)\right)\mu_{\mathrm{RTE}}^{\vec{b}}\left(y\right)dy+\int_{A}r_{2}\left(x\right)\mu \left(x\right)dx\\ & =\int_{A}\mu_{\mathrm{RTE}}^{\vec{b}}\left(y\right)dy\\ & =\mu_{\mathrm{RTE}}^{\vec{b}}\left(A\right)\;. \end{array}$$

 □

This theorem demonstrates that the high-resolution target distribution, for us $\mu_{\mathrm{RTE}}^{\vec{b}}$, is the invariant distribution of $P_{2}$. Thus, the two-level MCMC method gives a list of elements $\left\{x_{k}\right\}$ that can be regarded as samples from $\mu_{\mathrm{RTE}}^{\vec{b}}$.

To generate samples from the posterior based on the RTE, using the one-level MCMC method, for each new proposal, the forward map must be computed. To do this, one injects *K* boundary data $\phi_{k}$ and computes *J* outgoing data $l_{j}$, meaning that the RTE is solved *K* times and each time the solution is evaluated at *J* locations. As this is computationally expensive, one looks for a cheaper way to sample from the distribution. From Theorem 3, we have that in the diffusion limit, the posterior based on the RTE is close to the posterior based on the DE. The diffusion equation is significantly faster to solve because it is only over physical space. Therefore, in the strong scattering regime, we can save time on the computation by using the DE-assisted two-level MCMC method, in which the DE is used as a surrogate model to filter out bad draws. This saves computational effort spent on evaluating bad samples using the high-resolution model. The diffusion equation posterior can be used to reject bad samples, so that we have to evaluate the RTE posterior fewer times overall.

### 4.2. Properties of DE-Assisted Two-Level MCMC

In this section, we first present our result concerning the acceptance rate of the DE-assisted MCMC method and its dependence on ϵ, and then present the computational cost of our method compared to the one-level MCMC method.

#### 4.2.1. Acceptance Rate of DE-Assisted Two-Level MCMC

There are many ways to improve MCMC. Different sampling methods such as the Gibbs sampler or independence samplers can make the algorithm more efficient. There are also delayed acceptance/rejection methods, and adaptive methods in which the low-resolution model is improved at each stage using results from the high-resolution model [23]. However, this is not the focus of the current paper. Our method is a delayed acceptance method. It relies on the two-level MCMC algorithm and the diffusion limit to improve the efficiency of sampling from the posterior distribution for the RTE.

We emphasize that the DE-assisted two-level MCMC algorithm in Algorithm 3 has two acceptance rates, α and β. The first-level acceptance rate α depends only on the posterior based on the DE, so it can have no ϵ dependence. The second-level acceptance rate β, depends on ϵ through the posterior based on the RTE. As ϵ→0, the posterior based on the RTE becomes closer to the posterior based on the DE, so we expect that the samples that pass the selection criterion have a high chance of being accepted in the second step as well. We quantify this in the following proposition.

**Proposition** **2.** *In the diffusion limit, the acceptance rate β is high:*

(26) $$|\beta -1|=O(\epsilon^{1-\alpha })\;,$$

*as long as the proposal is reasonably close to the measured data,*

$$\begin{array}{c} ∥G_{\mathrm{DE}}\left(y\right)-\vec{b}{∥}_{2}\leq \gamma \sqrt{-\alpha \ln\epsilon }\;, \end{array}$$

*where γ is the variance of the noise and α is any constant between 0 and 1.*

**Proof.**  Suppose $∥G_{\mathrm{DE}}\left(y\right)-\vec{b}{∥}_{2}\leq \gamma \sqrt{-\alpha \ln\epsilon }$. Then, the likelihood function for the inverse diffusion equation is

$$\begin{array}{cc} \mu_{\mathrm{DE}}^{\sigma }\left(y\right) & =\exp\left(-\frac{1}{\gamma^{2}}{∥G^{\mathrm{DE}}\left(y\right)-\vec{b}∥}_{2}^{2}\right)>\exp\left(-\frac{1}{\gamma^{2}}\left(\gamma^{2}\left(-\alpha \ln\epsilon \right)\right)\right)\;. \end{array}$$

In other words,

$$\begin{array}{c} \mu_{\mathrm{DE}}^{\sigma }\left(y\right)>\epsilon^{\alpha }\;. \end{array}$$

Since the likelihood function is greater than ϵ and the prior has no ϵ dependence, the posterior distribution will also be greater than ϵ,

(27) $$\begin{array}{c} \mu_{\mathrm{DE}}^{\vec{b}}\left(y\right)=\mu_{0}\left(\sigma \right)\mu_{\mathrm{DE}}^{\sigma }\left(y\right)=O(\epsilon^{\alpha })\;. \end{array}$$

Considering the form of β in Equation (24), we have

(28) $$\begin{array}{cc} \beta & =\min\left\{1,\frac{\mu_{\mathrm{DE}}^{\vec{b}}\left(x\right)\mu_{\mathrm{RTE}}^{\vec{b}}\left(y\right)}{\mu_{\mathrm{DE}}^{\vec{b}}\left(y\right)\mu_{\mathrm{RTE}}^{\vec{b}}\left(x\right)}\right\}=\min\left\{1,\frac{\mu_{\mathrm{DE}}^{\vec{b}}\left(x\right)\left(\mu_{\mathrm{DE}}^{\vec{b}}\left(y\right)+C\left(y\right)\epsilon \right)}{\mu_{\mathrm{DE}}^{\vec{b}}\left(y\right)\left(\mu_{\mathrm{DE}}^{\vec{b}}\left(x\right)+C\left(x\right)\epsilon \right)}\right\}\;, \end{array}$$

using Theorem 3. Considering

$$\begin{array}{cc} \frac{\mu_{\mathrm{DE}}^{\vec{b}}\left(x\right)\left(\mu_{\mathrm{DE}}^{\vec{b}}\left(y\right)+C\left(y\right)\epsilon \right)}{\mu_{\mathrm{DE}}^{\vec{b}}\left(y\right)\left(\mu_{\mathrm{DE}}^{\vec{b}}\left(x\right)+C\left(x\right)\epsilon \right)} & =\frac{\mu_{\mathrm{DE}}^{\vec{b}}\left(x\right)\mu_{\mathrm{DE}}^{\vec{b}}\left(y\right)+\mu_{\mathrm{DE}}^{\vec{b}}\left(x\right)C\left(y\right)\epsilon }{\mu_{\mathrm{DE}}^{\vec{b}}\left(y\right)\mu_{\mathrm{DE}}^{\vec{b}}\left(x\right)+\mu_{\mathrm{DE}}^{\vec{b}}\left(y\right)C\left(x\right)\epsilon }\\ & =\frac{1+C\left(y\right)\epsilon /\mu_{\mathrm{DE}}^{\vec{b}}\left(y\right)}{1+C\left(x\right)\epsilon /\mu_{\mathrm{DE}}^{\vec{b}}\left(x\right)}\\ & =\left(1+\frac{C(y)\epsilon }{\mu_{\mathrm{DE}}^{\vec{b}}\left(y\right)}\right)\left(1-\frac{C(x)\epsilon }{\mu_{\mathrm{DE}}^{\vec{b}}\left(x\right)}+\cdots \right)\\ & =1+O(\epsilon^{1-\alpha })\;, \end{array}$$

where the Taylor expansion is possible due to Equation (27). Therefore, for small ϵ,

$$\begin{array}{c} |\beta -1|=O(\epsilon^{1-\alpha })\;. \end{array}$$

 □

This proposition demonstrates that as ϵ→0 and the posterior based on the RTE becomes closer to the posterior based on the DE, more and more samples are accepted at the second level.

#### 4.2.2. Computational Cost Comparison

In this section, we discuss the computational cost of our method compared to the one-level MCMC method.

**Proposition** **3.** *Let Cost${}_{1}$ denote the cost of obtaining k accepted samples using the one-level MCMC method, and Cost${}_{2}$ denote the cost of obtaining k accepted samples using the DE-assisted two-level MCMC method. Let $r_{1}$ denote the acceptance rate of the one-level MCMC method. Let α denote the first-level acceptance rate of the DE-assisted two-level MCMC method, and β denote the second-level acceptance rate of the method.*

- *Cost${}_{1}$ and Cost${}_{2}$ are*
  $$\begin{array}{cc} {Cost}_{1} & =\frac{k}{r_{1}}C_{R}\\ {Cost}_{2} & =\frac{k}{\alpha \beta }C_{D}+\frac{k}{\beta }C_{R}, \end{array}$$
  *where $C_{D}=O\left(N_{x}^{cd}\right)$ is the cost of solving the diffusion equation with $N_{x}$ grid points in d dimensions, where c is the cost of the linear algebra solver used. Similarly, $C_{R}=O\left(N_{x}^{cd}N_{v}^{c(d-1)}\right)$ is the cost of solving the radiative transfer equation with the same parameters, and $N_{v}$ is the number of grid points in the velocity domain.*
- *Considering $C_{R}\gg C_{D}$, we have*
  $$\begin{array}{c} \frac{{Cost}_{2}}{{Cost}_{1}}\approx \frac{r_{1}}{\beta }. \end{array}$$
*Thus, the cost saving of our method comes from $\beta >r_{1}$.*

**Proof.**  First, we examine the cost of evaluating the posterior distribution based on the diffusion equation compared to the posterior based on the radiative transfer equation. The cost of evaluating each posterior distribution is directly proportional to the cost of computing each solution to the equation, so we examine instead the cost of computing a solution to the diffusion equation and the radiative transfer equation. Considering $C_{D}=O\left(N_{x}^{cd}\right)$, and $C_{R}=O\left(N_{x}^{cd}N_{v}^{c(d-1)}\right)$, we see that the diffusion equation is significantly cheaper to compute than the radiative transfer equation, depending on the number of grid points in the velocity domain.

Next, we compute the number of MCMC iterations required to obtain *k* accepted samples for each method. For the one-level MCMC method, we simply have

$$\begin{array}{c} N_{1}r_{1}=k, \end{array}$$

where $r_{1}$ is the acceptance rate and $N_{1}$ is the total number of iterations considered. For the two-level method,

$$\begin{array}{c} N_{2}r_{2}=k, \end{array}$$

where $N_{2}$ is the total number of iterations, and $r_{2}$ is the overall acceptance rate. To be more specific, a single sample must go through both levels of evaluation in order to be accepted. Supposing that the acceptance rate at the first level is α, and the acceptance rate at the second level is β, then, the overall acceptance rate is $r_{2}=\alpha \beta$, which gives us

$$\begin{array}{c} N_{2}\alpha \beta =k. \end{array}$$

Using the above results, we can compute the cost of obtaining *k* accepted samples using the one-level MCMC method,

$$\begin{array}{c} {\mathrm{Cost}}_{1}=N_{1}C_{R}=\frac{k}{r_{1}}C_{R}. \end{array}$$

Similarly, the cost of obtaining *k* accepted samples using the two-level method is

(29) $$\begin{array}{c} {\mathrm{Cost}}_{2}=N_{2}C_{D}+\frac{k}{\beta }C_{R}=\frac{k}{\alpha \beta }C_{D}+\frac{k}{\beta }C_{R}. \end{array}$$

Considering $C_{D}\ll C_{R}$, the term containing $C_{D}$ may be dropped out of Equation (29). Then we have

$$\begin{array}{c} \frac{{\mathrm{Cost}}_{2}}{{\mathrm{Cost}}_{1}}=\frac{r_{1}}{\beta }. \end{array}$$

Then for $\beta \gg r_{1}$, the cost of obtaining *k* samples using the DE-assisted two-level MCMC is much less than the cost of obtaining *k* samples using the one-level MCMC. □

In practice, β and $r_{1}$ depend on the sampling strategy used, the initial values of the MCMC parameters, and the step size, so we give only a theoretical asymptotic estimate of the cost comparison.

## 5. Numerics

We summarize our numerical evidence in this section. We will first show the convergence in the forward setting, and demonstrate that the solution to the RTE indeed converges to that of the DE. We then demonstrate, with two different media configurations, the convergence of the posterior distribution using the Metropolis–Hastings MCMC method. The 2-level MCMC result will also be shown.

Throughout this section, the DE is computed using the standard finite element method, and the RTE solver is a preconditioned GMRES-based method with upwinding in the physical domain, designed in [29].

### 5.1. Forward Model Convergence

We first review the convergence in the forward setup. As discussed in Theorem 2, in the zero limit of ϵ, the solution to the RTE becomes the solution to the DE, which drives the convergence of the albedo operator to the DtN map. We show this numerically below.

We first use a pseudo-2D example, with the *y*-direction assumed to be homogeneous. The RTE is then a degenerate case given by

$$\begin{array}{c} \cos\theta \partial_{x}f=\sigma \left(x\right)Lf\;. \end{array}$$

**Figure 1:**
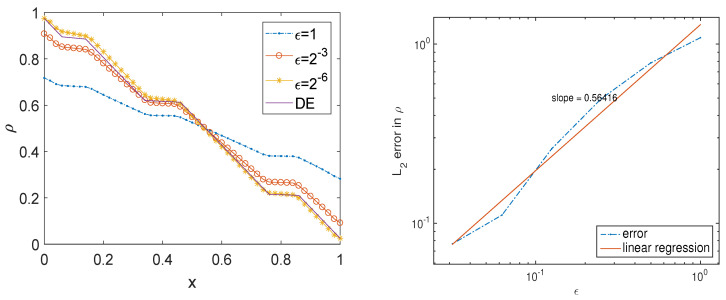
The solution ρ as a function of *x* for different ϵ, and the error in the solution ρ as a function of ϵ.

Here, the media is set as

$$\begin{array}{c} \sigma \left(x\right)=1+9\chi_{\left[0.05,0.15\right]}\left(x\right)+19\chi_{\left[0.35,0.45\right]}\left(x\right)+29\chi_{\left[0.75,0.85\right]}\left(x\right)\;, \end{array}$$

where $\chi_{\left[a,b\right]}$ is the characteristic function. Figure 1 contains a plot of the solution ρ as a function of *x* for the given σ for the RTE with $\epsilon =1,2^{-3},$ and $2^{-6}$, as well as the solution for the DE. Numerically, we set dx=0.05 and dv=2π/16. As ϵ becomes smaller and smaller, the density ρ of the radiative transfer equation becomes closer and closer to the solution of the diffusion equation. We plot the error on the right panel of Figure 1 on a log-log scale as a function of ϵ, measured in $L_{2}\left(dx\right)$. The values of the error are shown in Table 1.

**Figure 2:**
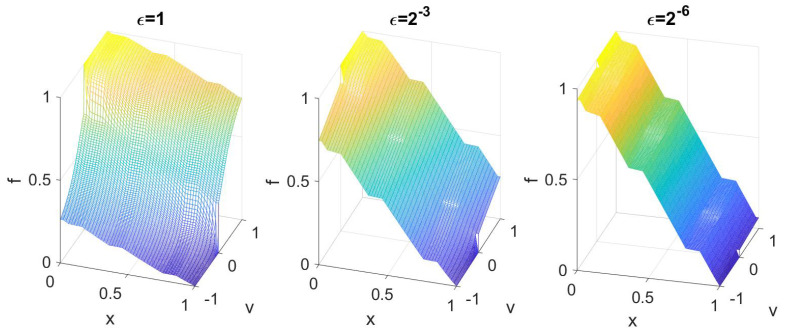
The solution as a function of *x* and *v* for different ϵ.

We furthermore plot the solution *f* as a function on the phase space. As shown in Theorem 2, in the diffusion limit, *f* loses its velocity dependence, and the solution becomes flat in *v*. This is seen numerically as well in Figure 2, which shows f(x,v) for different ϵ.

**Figure 3:**
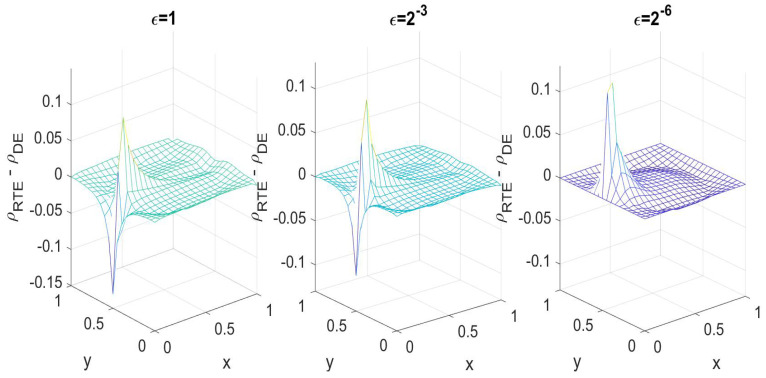
Difference between the RTE and DE solutions in 2D for different ϵ.

**Figure 4:**
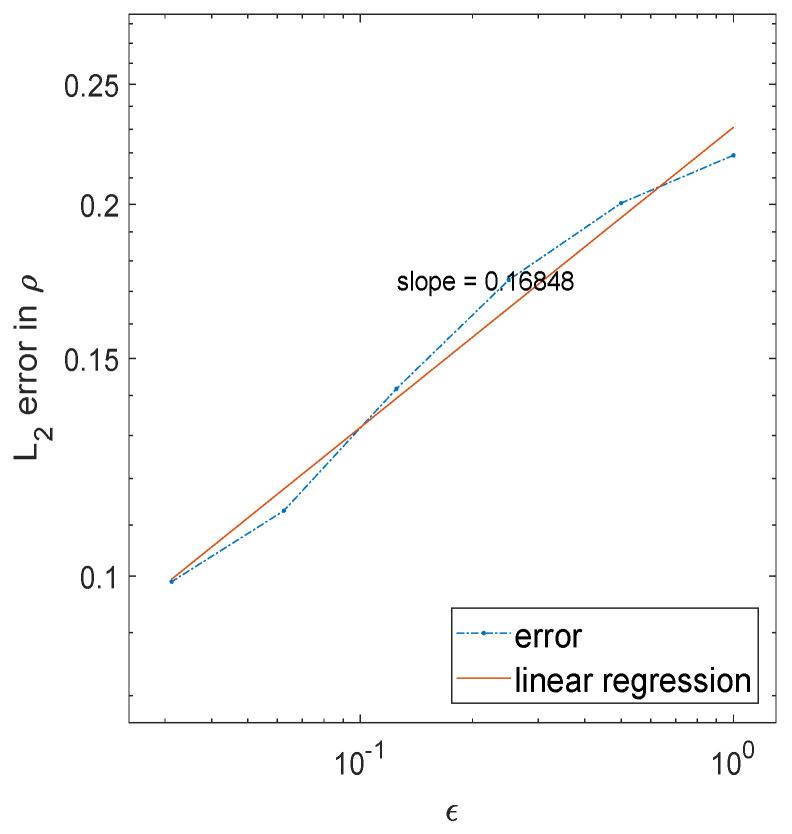
$L_{2}$ norm in the error for the 2D solution ρ as a function of ϵ.

Similar convergence is numerically evaluated in two dimensions where $\Omega ={\left[0,1\right]}^{2}$ and σ is chosen to be

$$\begin{array}{c} \sigma (x)=1+h\;\chi (|x-c|<r)\;, \end{array}$$

where $x=\left(x,y\right)\in {\left[0,1\right]}^{2}$, h=10 and r=0.4. χ is the characteristic function, and the center c=(0.5,0.5) is given. It is hard to visualize a 3D object, and we only plot the difference in ρ between the radiative transfer and diffusion solutions for different ϵ in Figure 3. We observe that as ϵ→0, the error decreases, as plotted in Figure 4.

### 5.2. Reconstruction Example 1

In this example, we test the inverse problem in $\Omega ={\left[0,1\right]}^{2}\subset R^{2}$ with the true media

$$\begin{array}{c} \sigma (x)=1+h\chi (|x-c|<r)\;, \end{array}$$

where r=0.4 and h=10, and c=(0.5,0.5). It represents a circle with radius *r* and height *h* in the middle of the unit square. Since the media configuration is uniquely determined by the two parameters *r* and *h*, the reconstructed posterior distribution then is a distribution function on the (r,h) domain. The convergence and the accuracy of the method is independent of dimension, but we need to use an example that can be visualized to the readers. Besides, in most application problems the bio-tissues to be reconstructed have two or three components that have very different optical properties, and the model used here is a realistic representation of the physical scenario. To compute the forward RTE, we use the GMRES method. We set dx=0.05 and dv=2π/16. The light sources are placed on the left boundary (x=0) for all discrete grids in *y*-space. This means 20 experiments are being looked at. For all these experiments, the sensors are located at all grid points on the boundaries. For each MCMC sample (r,h), to evaluate $\mu_{\mathrm{RTE}}^{\vec{b}}\left(r,h\right)$, 20 forward RTE solvers are run with the media configuration (r,h) with the boundary condition being Kronecker delta functions at each light source.

We briefly mention that to visualize the data, we do apply the kernel density estimator that smooths out the solution with a Gaussian kernel.

**Figure 5:**
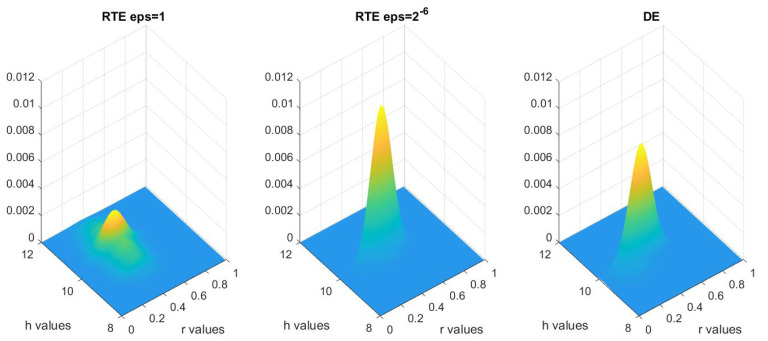
Multivariate kernel distribution representing the posterior distributions for DE, RTE with ϵ=1 and $\epsilon =2^{-6}$ obtained using the one-level MCMC method.

We first consider the solution to this problem in the one-level MCMC framework. The prior distribution is taken to be uniform in *r* from 0 to 0.5, and uniform in *h* from 8 to 12, and the variance of the noise is taken to be $10^{-4}$. With merely $10^{3}$ MCMC steps, one can already distinguish the distribution functions. As seen in Figure 5, the posterior distribution using the RTE model with ϵ=1 is much more smeared out than the one with $\epsilon =2^{-6}$, and the posterior distribution using the RTE model with $\epsilon =2^{-6}$ is significantly closer to that using the DE model.

To quantize the difference between these three distributions, we compute the Hellinger distances, as documented in Table 2, which numerically confirms Theorem 3.

**Figure 6:**
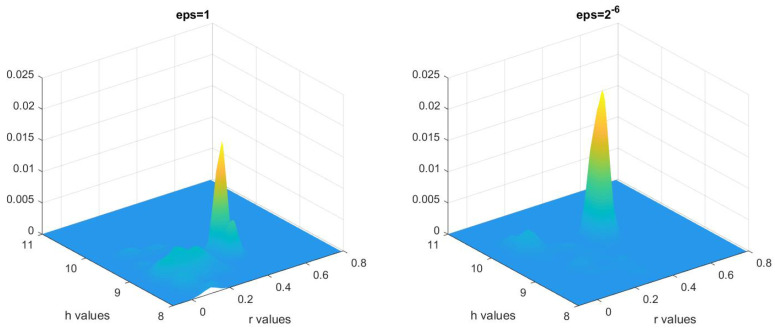
Multivariate kernel distribution representing posterior distribution obtained from the two-level MCMC method for ϵ=1 and $\epsilon =2^{-6}$.

We then study the two-level MCMC method with the same parameters. The Gaussian-kernel smoothed posterior distributions are shown in Figure 6.

We examined the acceptance rate β: the number of accepted particles on the second level versus the number of accepted particle on the first level. As suggested in Proposition 2, smaller ϵ should give a higher acceptance rate, and this is truly seen in numerics. The results are shown in Table 3.

We also expect for small ϵ, distributions computed using the one-level MCMC should be similar to the ones computed using DE-assisted method. To compare them, we compute the Hellinger distance between the distribution based on the RTE computed using one-level MCMC for $\epsilon =2^{-6}$, in Figure 5 and the distribution based on the RTE computed using the DE-assisted MCMC for $\epsilon =2^{-6}$, shown in Figure 6. We obtain

$$\begin{array}{c} d_{\mathrm{Hell}}\left(\mu_{\mathrm{RTE}}^{\vec{b}}{\left(\sigma \right)}_{1\text{-}\mathrm{level}},\mu_{\mathrm{RTE}}^{\vec{b}}{\left(\sigma \right)}_{\mathrm{DE}\text{-}\mathrm{assisted}}\right)=0.2289, \end{array}$$

demonstrating that the distributions based on the methods are similar.

Finally, we report the computational cost savings of using the DE-assisted two-level MCMC method compared to the one-level MCMC method. The values of β and $r_{1}$ are documented in Table 4. From the table, we see that using the DE-assisted two-level MCMC method saves us roughly 20% of the RTE evaluations, for each value of ϵ.

We emphasize that β and $r_{1}$ highly depend on the sampling strategy used, the initial values of the MCMC parameters, the step size and how the prior distribution looks. If the prior distribution is centered far away from the maximum likelihood point, a large amount of sample points are needed to give a fair presentation of the posterior distribution function, and the behavior of the underlying equation would be crucial in computational saving.

### 5.3. Reconstruction Example 2

In this example, we take a more complicated media σ:

$$\sigma \left(x\right)=1+\sum_{i=1}^{5}h_{i}\chi_{i}(|x-c_{i}\left|<r_{2}\right)\;,$$

where $x=\left(x,y\right)\in {\left[0,1\right]}^{2}$ and $c_{i}$ are given, and $\chi_{i}$ are characteristic functions. The list of height $h_{i}$ and radii $r_{i}$ are parameters to be reconstructed, and thus we have ten unknown parameters. Numerically, we set dx=0.05, dv=2π/16, and we place the light sources at the left boundary as in Example 1. Again, we take $10^{3}$ MCMC steps. As before, to compute the forward RTE, we use the GMRES method. The light sources are placed on the left boundary (x=0) for all discrete grids in *y*-space. This means that 20 experiments are being looked at. For all these experiments, the sensors are located at all grid points on the boundaries. For each MCMC sample (r,h), to evaluate $\mu_{\mathrm{RTE}}^{\vec{b}}\left(r,h\right)$, 20 forward RTE solvers are run with the media configuration (r,h) with the boundary condition being Kronecker delta functions at each light source.

The true parameters are set as

$$\begin{array}{c} \left(h_{1},r_{1}\right)=\left(5,0.19\right)\;,\;\left(h_{2},r_{2}\right)=\left(1,0.1\right)\;,\;\left(h_{3},r_{3}\right)=\left(7,0.09\right)\;,\\ \left(h_{4},r_{4}\right)=\left(4,0.13\right)\;,\;\left(h_{5},r_{5}\right)=\left(10,0.04\right), \end{array}$$

with the centers

$$\begin{array}{c} c_{1}=\left(0.5,0.5\right)\;,\;c_{2}=(0.2,\;0.35)\;,\;c_{3}=\left(0.75,0.2\right)\;,\\ c_{4}=\left(0.8,0.85\right)\;,\;c_{5}=\left(0.3,0.8\right)\;. \end{array}$$

**Figure 7:**
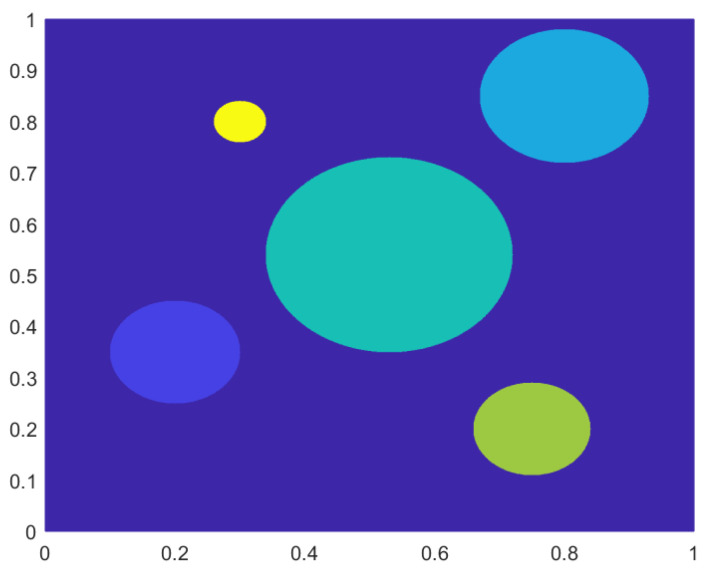
The true σ as a contour plot for Example 2.

The true σ is depicted in Figure 7.

**Figure 8:**
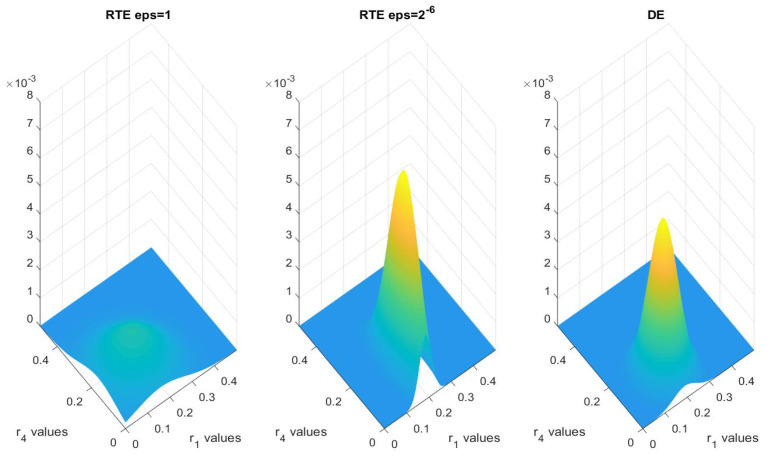
Multivariate kernel distribution for $r_{1}$ and $r_{4}$ for DE and RTE with ϵ=1 and $\epsilon =2^{-6}$ using one-level MCMC.

To test the one-level MCMC result, we feed the algorithm an initial guess that could be 40% away from the true value:

$$\begin{array}{c} h_{i}^{\mathrm{guess}}=\left(0.6+0.4\cdot \mathrm{rand}\right)\cdot h_{i}^{\mathrm{true}}\;, \end{array}$$

and similarly for each $r_{i}$. The prior is set as a uniform distribution, and the variance of the noise is again taken to be $10^{-4}$. In Figure 8, we plot the marginal posterior distribution of $\left(r_{1},r_{4}\right)$ of the DE, and RTE with ϵ=1, and $\epsilon =2^{-6}$. The posterior distribution of RTE with small ϵ is strikingly closer to the DE model, while the RTE model with big ϵ gives a very different result. In Table 5 we document the means and variances of the marginal distributions for $r_{1}$ and $r_{4}$.

We again compute the Hellinger distance between the two marginal distributions for $\left(r_{1},r_{4}\right)$ of the posterior distributions with respect to ϵ, and it is documented in Table 6. The results numerically confirm Theorem 3.

As done in Example 1, we document the acceptance rate. As seen clearly in Table 7, the acceptance rate increases as ϵ shrinks to zero.

**Figure 9:**
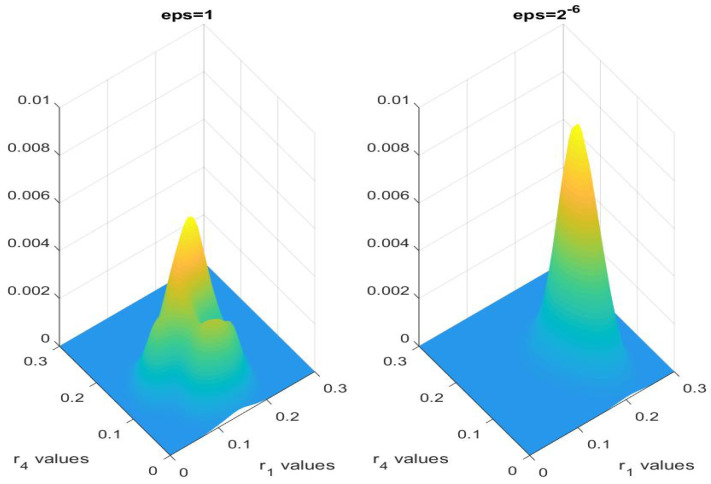
Multivariate kernel distribution for $r_{1}$ and $r_{4}$ using two-level MCMC with ϵ=1 and $\epsilon =2^{-6}$.

As before, we expect that the small ϵ distributions computed using the one-level MCMC case and using our method should be similar. To compare them, we compute the Hellinger distance between the distribution based on the RTE computed using one-level MCMC for $\epsilon =2^{-6}$, in Figure 8 and the distribution based on the RTE computed using the DE-assisted MCMC for $\epsilon =2^{-6}$, shown in Figure 9. We obtain

$$\begin{array}{c} d_{\mathrm{Hell}}\left(\mu_{\mathrm{RTE}}^{\vec{b}}{\left(\sigma \right)}_{1\text{-}\mathrm{level}},\mu_{\mathrm{RTE}}^{\vec{b}}{\left(\sigma \right)}_{\mathrm{DE}\text{-}\mathrm{assisted}}\right)=0.1357, \end{array}$$

again demonstrating that the distributions based on the methods are similar.

Finally, we report the computational cost savings of using the DE-assisted two-level MCMC method compared to the one-level MCMC method for Example 2. The values of β and $r_{1}$ for Example 2 are documented in Table 8. From the table, we see that using the DE-assisted two-level MCMC method saves us roughly 12% of the RTE evaluations, for both values of ϵ that we considered.

We again emphasize that β and $r_{1}$ highly depend on the sampling strategy used, the initial values of the MCMC parameters, and the step size.

## 6. Conclusions

In this paper, we solve the inverse problem for the RTE using Bayesian inference, which gives us a posterior distribution for the quantity of interest. Accessing concrete information about this distribution using MCMC can be difficult, because the RTE is fairly expensive to solve. However, in the strong scattering regime with the Knudsen number ϵ going to zero, the posterior distribution based on the RTE converges to the posterior distribution based on the DE. With this knowledge, we employ a two-level MCMC technique, in which the posterior based on the DE is used to create good samples for the posterior based on the RTE. In this way, we save time by rejecting poor samples using the posterior based on the DE distribution, which is fast to compute. This reduces the number of times we must solve the inverse problem for the RTE, which improves the efficiency of the computation. We also prove that the second-level acceptance rate of this method is close to one in the diffusion limit, meaning that samples accepted at the first level have a high chance of being accepted at the second level, if ϵ is small. 

## Figures and Tables

**Table 1:** The values of the error in the solution ρ as a function of ϵ.

ϵ	1	$\frac{1}{2}$	$\frac{1}{4}$	$\frac{1}{16}$	$\frac{1}{32}$	$\frac{1}{64}$
error	0.2191	0.2004	0.1737	0.1418	0.1130	0.0990

**Table 2:** Hellinger distance: $d_{\mathrm{Hell}}\left(\mu_{\mathrm{DE}}^{\vec{b}}\left(\sigma \right),\mu_{\mathrm{RTE}}^{\vec{b}}\left(\sigma \right)\right)$ with $\mu_{\mathrm{RTE}}^{\vec{b}}\left(\sigma \right)$ computed with RTE as the forward solver using ϵ=1, $\epsilon =2^{-3}$ and $\epsilon =2^{-6}$ respectively.

ϵ	1	$2^{-3}$	$2^{-6}$
$d_{\mathrm{Hell}}\left(\mu_{\mathrm{DE}}^{\vec{b}}\left(\sigma \right),\mu_{\mathrm{RTE}}^{\vec{b}}\left(\sigma \right)\right)$	0.6418	0.5322	0.2219

**Table 3:** Acceptance rate β for Example 1.

ϵ	1	$2^{-3}$	$2^{-6}$
acceptance rate	0.6931	0.8736	0.8939

**Table 4:** Computational cost comparison.

	ϵ=1	$\epsilon =2^{-6}$
β	0.6931	0.8939
$r_{1}$	0.6000	0.7350
$r_{1}/\beta$	0.8656	0.8222

**Table 5:** Means and variances of the marginal distributions for $r_{1}$ and $r_{4}$ for DE, RTE with ϵ=1, and RTE with $\epsilon =2^{-6}$ for two-level MCMC.

	Mean ($r_{1}$)	Mean ($r_{4}$)	Var ($r_{1}$)	Var ($r_{4}$)
RTE, ϵ=1	0.1483	0.1518	0.0468	0.0464
RTE, $\epsilon =2^{-6}$	0.1856	0.1278	0.042	0.0076
DE	0.1895	0.1273	0.0078	0.0038

**Table 6:** Hellinger distance for the marginal distributions for $r_{1}$ and $r_{4}$: $d_{\mathrm{Hell}}\left(\mu_{\mathrm{DE}}^{\vec{b}}\left(\sigma \right),\mu_{\mathrm{RTE}}^{\vec{b}}\left(\sigma \right)\right)$ with $\mu_{\mathrm{RTE}}^{\vec{b}}\left(\sigma \right)$ computed with RTE as the forward solver using ϵ=1, $\epsilon =2^{-3}$ and $\epsilon =2^{-6}$ respectively.

ϵ	1	$2^{-3}$	$2^{-6}$
$d_{\mathrm{Hell}}\left(\mu_{\mathrm{DE}}^{\vec{b}}\left(\sigma \right),\mu_{\mathrm{RTE}}^{\vec{b}}\left(\sigma \right)\right)$	0.8693	0.6324	0.4440

**Table 7:** Acceptance rate β for Example 2.

ϵ	1	$2^{-3}$	$2^{-6}$
acceptance rate	0.7230	0.8969	0.9305

**Table 8:** Computational cost comparison.

	ϵ=1	$\epsilon =2^{-6}$
β	0.7230	0.9305
$r_{1}$	0.6450	0.8180
$r_{1}/\beta$	0.8921	0.8791
